# Magnetic resonance imaging of the spine in a pediatric population: incidental findings

**DOI:** 10.1590/0100-3984.2018.0099

**Published:** 2020

**Authors:** Renato Tavares Daher, Murilo Tavares Daher, Ricardo Tavares Daher, Marcelo Fouad Rabahi, Marcos Rassi Fernandes, Hugo Pereira Pinto Gama

**Affiliations:** 1 Universidade Federal de Goiás (UFG), Goiânia, GO, Brazil.; 2 Centro de Reabilitação e Readaptação Dr. Henrique Santillo (CRER), Goiânia, GO, Brazil.; 3 CRD Medicina Diagnóstica, Goiânia, GO, Brazil.

**Keywords:** Spine, Magnetic resonance imaging, Child, Diagnosis, Coluna vertebral, Ressonância magnética, Criança, Diagnóstico

## Abstract

**Objective:**

To determine the prevalence of incidental findings on magnetic resonance imaging (MRI) scans of the cervical, thoracic and lumbar spine in a paediatric population.

**Materials and Methods:**

We evaluated 190 spinal MRI examinations of patients aged ≤ 18 years of age. The study included only patients for whom complete medical records were available and who underwent complete MRI examination of the cervical, thoracic or lumbar spine, including whole-spine sagittal T2-weighted sequences. Imaging findings not related to the symptom or indication for MRI were considered incidental findings.

**Results:**

Of the 190 MRI examinations evaluated, 110 were in women and 80 were in men. The mean age of the study population was 12.46 ± 3.68 years. The main clinical indications for MRI in the sample were lumbago, scoliosis, dorsalgia and cervicalgia. Incidental findings were detected in the cervical, thoracic and lumbar spine in 40 (21.05%), 26 (13.83%) and 43 (22.63%) of the patients, respectively. The most common were (in the cervical spine) reversal/correction of the normal curvature; (in the thoracic spine) intravertebral disc herniation (Schmorl’s node) and disc dehydration; and (in the lumbar spine) disc protrusion (12 cases), Schmorl’s node (5 cases) and spondylolysis (4 cases).

**Conclusion:**

Incidental findings on MRI of the spine are less common in the paediatric population than in the adult population. Nevertheless, careful clinical evaluation of paediatric patients with complaints of axial and radiating pain is necessary in order to determine the correlation between symptoms and imaging findings.

## Abstract (pt)

**Objetivo:**

Determinar a prevalência de achados incidentais em exames de ressonância magnética (RM) da coluna cervical, torácica e lombar em uma população pediátrica.

**Materiais e Métodos:**

Foram avaliados 190 exames de RM da coluna vertebral de pacientes com até 18 anos de idade. Foram incluídos pacientes submetidos a RM de algum segmento da coluna vertebral (cervical, torácica e lombar) com exame completo para análise (inclusive sagital T2 da coluna total) e prontuário médico devidamente preenchido. Foram considerados achados incidentais todas as alterações de imagem que não apresentavam qualquer relação com a indicação do exame ou sintoma do paciente.

**Resultados:**

Dos 190 exames de RM avaliados, 110 eram de pacientes do sexo feminino e 80 de pacientes do sexo masculino. A idade média da população em estudo era de 12,46 ± 3,68 anos. As principais indicações clínicas para realização do exame de RM na amostra estudada foram lombalgia, escoliose, dorsalgia e cervicalgia. Os achados incidentais encontrados na coluna cervical, torácica e lombar foram 40 (21,05%), 26 (13,83%) e 43 (22,63%), respectivamente. Os achados incidentais mais frequentes no segmento cervical foram inversão/retificação da curvatura cervical fisiológica, no segmento torácico foram hérnia discal intrassomática (nódulo de Schmorl), seguida de desidratação discal, e no segmento lombar foram encontrados 12 exames com protrusão discal, 5 com hérnia discal intrassomática (nódulo de Schmorl) e 4 com espondilólise.

**Conclusão:**

A frequência de achados incidentais nos exames de RM da coluna vertebral na população pediátrica é menor do que na população adulta, sendo necessária avaliação clínica cuidadosa dos pacientes com queixa de dor axial e irradiada, para se estabelecer a estreita relação dos sintomas com os achados de imagem.

## INTRODUCTION

Magnetic resonance imaging (MRI) is the most significant technological advancement in the diagnostic examination of the spine. Because this diagnostic method is sensitive but not specific, asymptomatic individuals can present changes on MRI^(1,2)^, such changes being considered incidental findings. Knowledge of the characteristics of this diagnostic method is essential for the therapeutic management of patients undergoing MRI of the spine, particularly those with pain, because correlating symptoms with MRI findings is often difficult^(1)^.

In the adult population, the prevalence of incidental findings on MRI of the spine is high, particularly on scans of the lumbar spine^(1,2)^. However, few studies have evaluated the prevalence of incidental findings on MRI scans of the cervical and thoracic spine^(3)^.

A complaint of back pain, particularly in the lumbar region, is not uncommon in the paediatric population^(4)^, and many paediatric patients undergo MRI to identify the cause of the symptoms. However, there have been only a few studies evaluating the prevalence of incidental findings on MRI of the spine in this population^(5)^, and none of those studies have investigated the topic in the Brazilian population. The objective of the present study was to determine the prevalence of incidental findings on MRI of the cervical, thoracic and lumbar spine in a paediatric population in Brazil.

## MATERIALS AND METHODS

This was a retrospective analysis of spinal MRI examinations performed in patients ≤ 18 years of age at a tertiary-care hospital between October 2012 and February 2016. The study was approved by the local institutional review board (Reference no. 50999715.5.0000.0023). Because of the retrospective nature of the study, the requirement for written informed consent was waived.

Patients who underwent comprehensive MRI examination of the cervical, thoracic or lumbar spine were included in the study if their medical records were complete. Patients with neurofibromatosis, Marfan syndrome or neoplasia were excluded.

**Figure 1:**
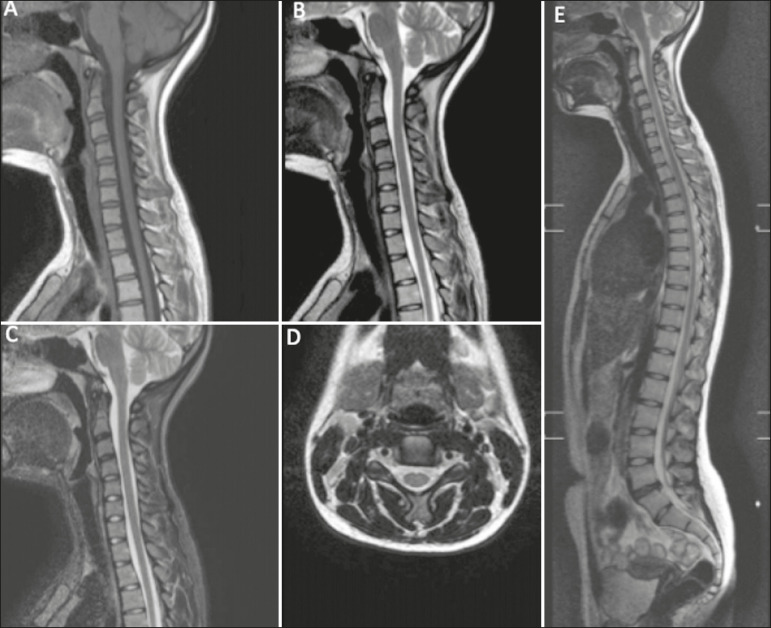
**A,B,C:** Sagittal T1-weighted, T2-weighted and T2-weighted STIR sequences of the cervical spine, respectively. **D:** Axial T2-weighted sequence of the cervical spine. **E:** Whole-spine sagittal T2-weighted sequence, including the base of the skull.

All patients were examined in a 1.5-T MRI scanner (Magnetom Avanto; Siemens Healthcare, Erlangen, Germany), with the following protocols:

- Cervical spine: sagittal T1-weighted sequence with a repetition time/echo time (TR/TE) of 400.0/6.2 ms; sagittal T2-weighted sequence (TR/TE of 300.0/100.0 ms); sagittal T2-weighted short-tau inversion-recovery (STIR) sequence (TR/TE of 2500.0/60.0 ms); axial T2-weighted sequence (TR/TE of 4150.0/120.0 ms); and whole-spine sagittal T2-weighted sequence (TR/TE of 2000.0/100.0 ms) including the base of the skull (Figure 1).

- Thoracic spine: sagittal T1-weighted sequence (TR/TE of 400.0/6.2 ms); sagittal T2-weighted sequence (TR/TE of 3000.0/100.0 ms); sagittal T2-weighted STIR sequence (TR/TE of 2500.0/60.0 ms); axial T2-weighted sequence (TR/TE of 2300.1/80.0 ms); and whole-spine sagittal T2-weighted sequence (TR/TE of 2000.0/100.0 ms) including the base of the skull.

- Lumbar spine: sagittal T1-weighted sequence (TR/TE of 400.0/8.0 ms); sagittal T2-weighted sequence (TR/TE of 2475.0/108.8 ms); axial T2-weighted sequence (TR/TE of 2704.1/100.0 ms); coronal T2-weighted STIR sequence (TR/TE of 2500.0/80.0 ms);; and whole-spine sagittal T2-weighted sequence (TR/TE of 2000.0/100.0 ms) including the base of the skull, as well as the cervical, thoracic and lumbar spine.

If vertebral deformity in the coronal plane was observed in any spinal segment, a coronal T2-weighted sequence (TR/TE of 2500.0/80.0 ms) was also analysed.

The examinations were evaluated by a radiologist, and the medical records were reviewed by an orthopaedic surgeon specialising in spinal surgery. These images were considered our normal sample, because it would be impractical to involve asymptomatic subjects (as is done in studies of adults), given the need to obtain informed consent from the parents/legal guardians and to perform the examination under anaesthesia.

Imaging findings that were not related to the indication for the examination or to a symptom, such as dilatation of the ependymal canal in a patient with a history of cervicobrachialgia, were classified as incidental findings. As whole-spine T2-weighted images were acquired for all patients, a patient with low back pain, for example, could be evaluated on the basis of the images of the cervical and thoracic spine. Discopathy was not considered an incidental finding, even if the main symptom was radiculopathy and not axial pain, because paediatric patients have difficulty differentiating between radiating pain and axial pain. Although some patients had more than one incidental finding, each alteration was considered in isolation because the objective of the study was to investigate the frequency of the individual findings.

A database was built with Microsoft Excel, and the data were analysed using the software Stata SE, version 12.0 (Stata Corp., College Station, TX, USA). The Shapiro-Wilk test was used in order to analyse the normality of continuous variables, and the variables are expressed as means and standard deviations. Pearson’s chi-square test was used for categorical variables, and the Student’s *t*-test was used for continuous variables. Values of *p* < 0.05 were considered statistically significant for all tests.

## RESULTS

A total of 190 MRI examinations of the spine were evaluated. Of those, 110 (57.89%) were performed in female patients and 80 (42.11%) were performed in male patients. The mean age of the study population was 12.46 ± 3.68 years.

The number of MRI examinations of the cervical, thoracic and lumbar spine requested was 24, 64 and 102, respectively (Table 1). As can be seen in Table 2, the main clinical indications for MRI in the study sample were lumbago (in 36.32%), scoliosis (in 22.63%), dorsalgia (in 15.79%) and cervicalgia (in 6.84%). The numbers of incidental findings in the cervical, thoracic and lumbar spine were 40 (21.05%), 26 (13.83%) and 43 (22.63%), respectively (Table 3). As shown in Table 4, the most common incidental finding in the cervical spine was reversal/correction of the normal curvature (33 cases; 17.37%).

**Table 1:** Characterisation of the study sample of spinal MRI examinations, by spinal segment.

		Female			Male			Age
Spinal segment	N	N	(%)		N	(%)		Mean ± SD
Cervical	24	15	(13.64)		9	(11.25)		11.25 ± 4.43
Thoracic	64	37	(33.63)		27	(33.75)		12.55 ± 2.96
Lumbar	102	58	(52.73)		44	(55.00)		12.62 ± 3.88

SD, standard deviation.

**Table 2:** Clinical indications for MRI in the study sample.

Clinical indication	N	(%)
Lumbago	69	(36.32
Scoliosis	43	(22.63)
Dorsalgia	30	(15.79)
Cervicalgia	13	(6.84)
Scoliosis and dorsalgia	4	(2.11)
Malformation*	4	(2.11)
Inability to walk	3	(1.58)
Delay in walking	3	(1.58)
Cervicalgia and lumbago	2	(1.05)
Dorsalgia and lumbago	2	(1.05)
Discitis	2	(1.05)
Transverse myelitis	1	(0.53)
Thoracic kyphosis	1	(0.53)
Total	190	(100.00)

* Suspicion of meningocele, meningocele + congenital deformity or congenital spondylolisthesis.

**Table 3:** Prevalence of incidental findings on MRI in the study population, by spinal segment.

Spinal segment	N	(%)
Cervical		
No incidental findings	150	(78.95)
Incidental findings	40	(21.05)
Thoracic		
No incidental findings	164	(86.32)
Incidental findings	26	(13.68)
Lumbar		
No incidental findings	147	(77.37)
Incidental findings	43	(22.63)

**Table 4:** Types of incidental findings on MRI in the study population, by spinal segment.

Incidental findings	N	(%)
In the cervical spine		
None	150	(78.95)
Reversal/correction of normal spinal curvature	33	(17.37)
Ectasia/dilatation of the ependymal canal	2	(1.05)
Disc dehydration	2	(1.05)
Disc protrusion + fissure of the annulus fibrosus	1	(0.53)
Changes in the spinal cord signal intensity	1	(0.53)
Ectasia/dilatation of the ependymal canal + reversal of the normal spinal curvature		
In the thoracic spine		
None	164	(86.32)
Intravertebral disc herniation (Schmorl's node)	10	(5.26)
Disc dehydration	4	(2.11)
Ectasia/dilatation of the ependymal canal	3	(1.58)
Disc protrusion	2	(1.06)
Calcified extruded disc	1	(0.53)
Vertebral fracture	1	(0.53)
Modic type 1 changes in the signal intensity of the vertebral endplate		
Disc dehydration + intravertebral disc herniation (Schmorl's node)		
Disc extrusion	1	(0.53)
Disc dehydration + intravertebral disc herniation (Schmorl's node)		
Osteophytes	1	(0.53)
In the lumbar spine		
None	147	(77.37)
Disc protrusion	12	(6.32)
Intravertebral disc herniation (Schmorl's node)	5	(2.63)
Spondylolysis	4	(2.11)
Spondylolysis + grade I spondylolisthesis	3	(1.58)
Disc extrusion	3	(1.58)
Disc dehydration + disc protrusion	3	(1.58)
Disc bulge	2	(1.05)
Disc dehydration	2	(1.05)
Migration of extruded disc	1	(0.53)
Disc protrusion + annulus fibrosus + disc dehydration	1	(0.53)
Sacralisation of the lumbar vertebra	1	(0.53)
Spondylolysis + disc bulge	1	(0.53)
Disc protrusion + acute intravertebral disc herniation (Schmorl's node)		
Facet joint osteoarthrosis + facet synovial cyst external to the vertebral canal + intravertebral disc herniation (Schmorl's node)		
Disc extrusion + disc protrusion	2	(1.05)

As the examination is performed with the patient in the supine position, we believe that it is not the ideal method to analyse the axes of the spine. The most common incidental findings in the thoracic spine were intravertebral disc herniation, also known as Schmorl’s node (10 cases; 5.26%), and disc dehydration (4 cases; 2.11%). In the lumbar spine, the most common incidental findings were disc protrusion (12 cases; 6.32%), intravertebral disc herniation-Schmorl’s node-(5 cases; 2.63%) and spondylolysis (4 cases; 2.11%) (Table 4).

Most (62.50%) of the incidental findings in the cervical spine were detected in females, whereas most (57.69%) of those in the thoracic spine were observed in males. In the lumbar spine, 53.48% of the incidental findings were detected in females. There were no significant differences between females and males in terms of the prevalence of incidental findings in the different spine segments (Table 5). However, as can be seen in Table 6, there was a significant difference between the patients with and without incidental findings in the lumbar spine, in terms of the mean age (*p* = 0.007).

**Table 5:** Incidental findings on MRI, by sex and spinal segment.

	Sex		
	Female	Male	
Spinal segment	N	N	*P*-value*
Cervical	25	15	0.514
Thoracic	11	15	0.081
Lumbar	23	20	0.497

* Pearson's chi-square test.

**Table 6:** Incidental findings on MRI, by mean age and spinal segment.

	Age		
Spinal segment	Without findings Mean ± SD	With findings Mean ± SD	*P*-value*
Cervical	12.40 ± 3.73	12.62 ± 3.61	0.732
Thoracic	12.30 ± 3.81	13.35 ± 2.88	0.183
Lumbar	12.05 ± 4.02	13.77 ± 1.82	0.007

* Student's-t test. SD, standard deviation.

## DISCUSSION

Previous studies have reported the prevalence of incidental findings in the cervical and lumbar spine in adult populations^(1,2,6-8)^. Although some studies have evaluated incidental findings in paediatric populations^(5,9)^, none of those studies were conducted in Brazil.

Ramadorai et al.^(5)^ found the prevalence of incidental findings in the cervical, thoracic and lumbar spine of paediatric patients to be 4.7%, 8.0% and 9.4%, respectively. In the present study, those rates were 21.05%, 13.83% and 22.63%, respectively. The fact that the proportion of incidental findings in the cervical spine in our sample was higher than that reported in the literature is attributable to the cases of inversion of the physiological lordosis observed in most of the examinations.

In a prospective study correlating MRI findings with the presence of lumbar pain in 13-year-old patients in Denmark^(9)^, the prevalence of incidental findings was found to be relatively high (approximately one-third of the patients had some degree of disc degeneration). The main findings associated with the presence of symptoms in that study were disc herniation, anterolisthesis, annular fissure, changes in the signal intensity of the endplates and loss of disc signal intensity.

Boden et al.^(2)^ evaluated lumbar MRI scans of 67 entirely asymptomatic adult subjects with no history of spinal disease. Among the subjects < 60 years of age, 20% had a herniated nucleus pulposus and one presented spinal stenosis; among those ≥ 60 years of age, 36% had disc herniations and 21% had spinal stenosis; and 35% of those 20-39 years of age showed some degree of disc degeneration or diffuse disc bulge. In another study, involving 63 asymptomatic individuals, Boden et al.^(7)^ identified incidental findings in the cervical spine in 19%. Among the individuals under 40 years of age, 10% had a herniated disc, 4% had foraminal stenosis and 25% showed some degree of disc degeneration or decrease in disc height. Among those ≥ 40 years of age, 5% had a herniated disc, 3% had disc bulges and 20% had foraminal stenosis.

Incidental findings are less common in the paediatric population than in the adult population. Nevertheless, clinical assessment of patients with axial or radiating skeletal pain is essential, and the correlation between symptoms and imaging findings should be established before treatment^(7,10,11)^.

One of the limitations of the present study was that only the whole-spine sagittal T2-weighted sequence was used in order to evaluate the spinal segments that were not within the scope of the examination. Performing prospective studies in paediatric populations is difficult because of the need for sedation during MRI examinations.

## CONCLUSION

The frequency of incidental findings on MRI examinations of the spine is lower in the paediatric population than in the adult population. Nevertheless, careful clinical evaluation of paediatric patients with complaints of axial and radiating pain is necessary in order to establish the correlation between symptoms and imaging findings. Determining the prevalence of and characterising such findings in the paediatric population may facilitate the assessment of their relevance and guide appropriate practices in asymptomatic children, as well as in children with borderline clinical conditions and radiological changes.

## References

[1] Jensen MC, Brant-Zawadzki MN, Obuchowski N (1994). Magnetic resonance imaging of the lumbar spine in people without back pain. N Engl J Med.

[2] Boden SD, Davis DO, Dina TS (1990). Abnormal magnetic-resonance scans of the lumbar spine in asymptomatic subjects. A prospective investigation. J Bone Joint Surg Am.

[3] Giuliano V, Giuliano C, Pinto F (2002). The use of flexion and extension MR in the evaluation of cervical spine trauma: initial experience in 100 trauma patients compared with 100 normal subjects. Emerg Radiol.

[4] Burton AK, Clarke RD, McClune TD (1996). The natural history of low back pain in adolescents. Spine (Phila Pa 1976).

[5] Ramadorai UE, Hire JM, DeVine JG (2014). Magnetic resonance imaging of the cervical, thoracic, and lumbar spine in children: spinal incidental findings in pediatric patients. Global Spine J.

[6] Deyo RA, Weinstein JN (2001). Low back pain. N Engl J Med.

[7] Boden SD, McCowin PR, Davis DO (1990). Abnormal magnetic-resonance scans of the cervical spine in asymptomatic subjects. A prospective investigation. J Bone Joint Surg Am..

[8] Boden SD (1996). The use of radiographic imaging studies in the evaluation of patients who have degenerative disorders of the lumbar spine. J Bone Joint Surg Am..

[9] Kjaer P, Leboeuf-Yde C, Sorensen JS (2005). An epidemiologic study of MRI and low back pain in 13-year-old children. Spine (Phila Pa 1976).

[10] Chou D, Samartzis D, Bellabarba C (2011). Degenerative magnetic resonance imaging changes in patients with chronic low back pain: a systematic review. Spine (Phila Pa 1976).

[11] Graves JM, Fulton-Kehoe D, Jarvik JG (2012). Early imaging for acute low back pain: one-year health and disability outcomes among Washington State workers. Spine (Phila Pa 1976).

